# Supplementary material for: Modeling glioblastoma heterogeneity as a dynamic network of cell states
Source: Mol Syst Biol. 2021 Sep 16;17(9):e10105. doi: 10.15252/msb.202010105 (PMC8444284; doi:10.15252/msb.202010105)
Supplement: Supplementary file 6 — Source Data for Figure 5 [file MSB-17-e10105-s004.zip › Figure5A_sourcedata/GSEA_3017/nosigdb_stateE.GseaPreranked.1622795031093/DOROSZKO_U3054MG_CLUSTER_4.html]

Details for gene set DOROSZKO\_U3054MG\_CLUSTER\_4[GSEA]

|  || Dataset | state63017 |
| Phenotype | NoPhenotypeAvailable |
| Upregulated in class | na\_neg |
| GeneSet | DOROSZKO\_U3054MG\_CLUSTER\_4 |
| Enrichment Score (ES) | -0.65693605 |
| Normalized Enrichment Score (NES) | -3.9444878 |
| Nominal p-value | 0.0 |
| FDR q-value | 0.0 |
| FWER p-Value | 0.0 |
Table: GSEA Results Summary

  

Fig 1: Enrichment plot: DOROSZKO\_U3054MG\_CLUSTER\_4      
 Profile of the Running ES Score & Positions of GeneSet Members on the Rank Ordered List

  

| PROBE | GENE SYMBOL | GENE\_TITLE | RANK IN GENE LIST | RANK METRIC SCORE | RUNNING ES | CORE ENRICHMENT || 1 | PEG10 |  |  | 54 | 0.506 | -0.0841 | No |
| 2 | DONSON |  |  | 274 | -0.251 | -0.4354 | No |
| 3 | SUPT16H |  |  | 276 | -0.251 | -0.4356 | No |
| 4 | MCMBP |  |  | 280 | -0.253 | -0.4390 | No |
| 5 | TUBA1C |  |  | 282 | -0.253 | -0.4392 | No |
| 6 | DRAXIN |  |  | 285 | -0.254 | -0.4411 | No |
| 7 | FIGNL1 |  |  | 290 | -0.257 | -0.4461 | No |
| 8 | RMI2 |  |  | 292 | -0.257 | -0.4463 | No |
| 9 | H2AFY |  |  | 297 | -0.257 | -0.4513 | No |
| 10 | YEATS4 |  |  | 300 | -0.258 | -0.4531 | No |
| 11 | RNF168 |  |  | 301 | -0.258 | -0.4516 | No |
| 12 | WDR34 |  |  | 303 | -0.258 | -0.4518 | No |
| 13 | HMGA1 |  |  | 313 | -0.261 | -0.4648 | No |
| 14 | AHCY |  |  | 317 | -0.262 | -0.4682 | No |
| 15 | PSIP1 |  |  | 319 | -0.263 | -0.4684 | No |
| 16 | TUBG1 |  |  | 323 | -0.263 | -0.4717 | No |
| 17 | CNTLN |  |  | 326 | -0.264 | -0.4735 | No |
| 18 | MAGOHB |  |  | 328 | -0.264 | -0.4736 | No |
| 19 | NCAPH2 |  |  | 331 | -0.264 | -0.4754 | No |
| 20 | RAD51C |  |  | 333 | -0.264 | -0.4755 | No |
| 21 | PAICS |  |  | 336 | -0.265 | -0.4773 | No |
| 22 | MCM5 |  |  | 337 | -0.265 | -0.4758 | No |
| 23 | KNTC1 |  |  | 352 | -0.269 | -0.4968 | No |
| 24 | NUDT15 |  |  | 356 | -0.270 | -0.5002 | No |
| 25 | CENPP |  |  | 366 | -0.273 | -0.5132 | No |
| 26 | POLE3 |  |  | 367 | -0.273 | -0.5116 | No |
| 27 | CDC25A |  |  | 368 | -0.273 | -0.5101 | No |
| 28 | POP7 |  |  | 370 | -0.273 | -0.5102 | No |
| 29 | NUDT8 |  |  | 376 | -0.275 | -0.5167 | No |
| 30 | TMEM237 |  |  | 383 | -0.276 | -0.5249 | No |
| 31 | SLBP |  |  | 386 | -0.277 | -0.5265 | No |
| 32 | NFATC2IP |  |  | 397 | -0.283 | -0.5411 | No |
| 33 | DERA |  |  | 402 | -0.284 | -0.5459 | No |
| 34 | LSM4 |  |  | 406 | -0.284 | -0.5492 | No |
| 35 | PPIH |  |  | 407 | -0.284 | -0.5476 | No |
| 36 | NASP |  |  | 410 | -0.285 | -0.5493 | No |
| 37 | ERCC6L |  |  | 421 | -0.290 | -0.5637 | No |
| 38 | CDK2 |  |  | 422 | -0.290 | -0.5621 | No |
| 39 | MYBL1 |  |  | 432 | -0.294 | -0.5750 | No |
| 40 | ZNF738 |  |  | 437 | -0.295 | -0.5798 | No |
| 41 | HMGXB4 |  |  | 438 | -0.296 | -0.5782 | No |
| 42 | JPT1 |  |  | 439 | -0.296 | -0.5765 | No |
| 43 | PPIF |  |  | 442 | -0.297 | -0.5781 | No |
| 44 | CDC7 |  |  | 451 | -0.299 | -0.5893 | No |
| 45 | CHTF18 |  |  | 455 | -0.301 | -0.5925 | No |
| 46 | MCM4 |  |  | 457 | -0.301 | -0.5924 | No |
| 47 | TRIM28 |  |  | 460 | -0.304 | -0.5939 | No |
| 48 | DNMT1 |  |  | 466 | -0.304 | -0.6003 | No |
| 49 | CLN6 |  |  | 470 | -0.305 | -0.6034 | No |
| 50 | SMC1A |  |  | 472 | -0.307 | -0.6033 | No |
| 51 | POLR3K |  |  | 474 | -0.307 | -0.6032 | No |
| 52 | CENPS |  |  | 476 | -0.307 | -0.6031 | No |
| 53 | UPF3B |  |  | 479 | -0.308 | -0.6046 | No |
| 54 | EXOSC9 |  |  | 481 | -0.308 | -0.6045 | No |
| 55 | NOP56 |  |  | 483 | -0.309 | -0.6044 | No |
| 56 | RSRC1 |  |  | 494 | -0.312 | -0.6188 | No |
| 57 | NUP205 |  |  | 495 | -0.313 | -0.6171 | No |
| 58 | RFC2 |  |  | 498 | -0.313 | -0.6186 | No |
| 59 | CBFB |  |  | 500 | -0.314 | -0.6184 | No |
| 60 | GGH |  |  | 504 | -0.314 | -0.6215 | No |
| 61 | SRRT |  |  | 509 | -0.317 | -0.6262 | No |
| 62 | BARD1 |  |  | 510 | -0.318 | -0.6244 | No |
| 63 | PGP |  |  | 512 | -0.318 | -0.6243 | No |
| 64 | BRI3BP |  |  | 515 | -0.319 | -0.6257 | No |
| 65 | HAUS1 |  |  | 518 | -0.320 | -0.6272 | No |
| 66 | POLA2 |  |  | 521 | -0.320 | -0.6286 | No |
| 67 | NUP62 |  |  | 529 | -0.323 | -0.6381 | No |
| 68 | ACTL6A |  |  | 533 | -0.325 | -0.6411 | No |
| 69 | RFC5 |  |  | 534 | -0.326 | -0.6393 | No |
| 70 | FKBP5 |  |  | 536 | -0.328 | -0.6391 | No |
| 71 | GPN3 |  |  | 537 | -0.328 | -0.6373 | No |
| 72 | PRIM1 |  |  | 539 | -0.328 | -0.6370 | No |
| 73 | TPRKB |  |  | 540 | -0.329 | -0.6352 | No |
| 74 | CEP57 |  |  | 545 | -0.330 | -0.6398 | No |
| 75 | MSH2 |  |  | 548 | -0.332 | -0.6412 | No |
| 76 | PLK4 |  |  | 551 | -0.334 | -0.6426 | No |
| 77 | MCM3 |  |  | 560 | -0.340 | -0.6536 | No |
| 78 | HAT1 |  |  | 561 | -0.340 | -0.6517 | No |
| 79 | RAD1 |  |  | 564 | -0.341 | -0.6530 | No |
| 80 | SASS6 |  |  | 567 | -0.342 | -0.6543 | No |
| 81 | TCF19 |  |  | 569 | -0.342 | -0.6540 | No |
| 82 | CCNE1 |  |  | 571 | -0.344 | -0.6537 | No |
| 83 | CKLF |  |  | 574 | -0.347 | -0.6550 | Yes |
| 84 | CCDC15 |  |  | 576 | -0.347 | -0.6547 | Yes |
| 85 | HMGB3 |  |  | 577 | -0.348 | -0.6528 | Yes |
| 86 | CENPX |  |  | 578 | -0.348 | -0.6508 | Yes |
| 87 | MIF |  |  | 580 | -0.349 | -0.6505 | Yes |
| 88 | RCC1 |  |  | 582 | -0.350 | -0.6502 | Yes |
| 89 | GEN1 |  |  | 584 | -0.351 | -0.6498 | Yes |
| 90 | NUP107 |  |  | 586 | -0.352 | -0.6495 | Yes |
| 91 | CACYBP |  |  | 589 | -0.352 | -0.6507 | Yes |
| 92 | DDX39A |  |  | 590 | -0.353 | -0.6488 | Yes |
| 93 | CDK5RAP2 |  |  | 591 | -0.353 | -0.6468 | Yes |
| 94 | EIF4EBP1 |  |  | 592 | -0.353 | -0.6449 | Yes |
| 95 | CMSS1 |  |  | 593 | -0.353 | -0.6429 | Yes |
| 96 | HSPB11 |  |  | 594 | -0.354 | -0.6409 | Yes |
| 97 | GGCT |  |  | 598 | -0.356 | -0.6438 | Yes |
| 98 | ZNF519 |  |  | 600 | -0.356 | -0.6434 | Yes |
| 99 | COMMD4 |  |  | 601 | -0.358 | -0.6414 | Yes |
| 100 | TTF1 |  |  | 602 | -0.358 | -0.6394 | Yes |
| 101 | HIRIP3 |  |  | 603 | -0.359 | -0.6374 | Yes |
| 102 | ZWILCH |  |  | 604 | -0.360 | -0.6354 | Yes |
| 103 | FANCB |  |  | 606 | -0.361 | -0.6350 | Yes |
| 104 | PRIM2 |  |  | 607 | -0.362 | -0.6330 | Yes |
| 105 | ASRGL1 |  |  | 608 | -0.362 | -0.6310 | Yes |
| 106 | PARP2 |  |  | 609 | -0.362 | -0.6290 | Yes |
| 107 | WDHD1 |  |  | 610 | -0.363 | -0.6270 | Yes |
| 108 | POC1A |  |  | 611 | -0.363 | -0.6250 | Yes |
| 109 | KIF18B |  |  | 612 | -0.363 | -0.6230 | Yes |
| 110 | TOPBP1 |  |  | 614 | -0.364 | -0.6226 | Yes |
| 111 | BTG3 |  |  | 615 | -0.365 | -0.6205 | Yes |
| 112 | RFWD3 |  |  | 617 | -0.365 | -0.6201 | Yes |
| 113 | ZGRF1 |  |  | 620 | -0.367 | -0.6213 | Yes |
| 114 | DDX11 |  |  | 622 | -0.370 | -0.6208 | Yes |
| 115 | MRE11 |  |  | 623 | -0.371 | -0.6188 | Yes |
| 116 | MTHFD2 |  |  | 625 | -0.372 | -0.6183 | Yes |
| 117 | WDR76 |  |  | 626 | -0.373 | -0.6163 | Yes |
| 118 | EXOSC8 |  |  | 630 | -0.376 | -0.6190 | Yes |
| 119 | SAMD1 |  |  | 632 | -0.377 | -0.6185 | Yes |
| 120 | RNASEH2A |  |  | 633 | -0.378 | -0.6164 | Yes |
| 121 | PPP2R3B |  |  | 634 | -0.380 | -0.6143 | Yes |
| 122 | RMI1 |  |  | 637 | -0.381 | -0.6154 | Yes |
| 123 | PHIP |  |  | 643 | -0.384 | -0.6213 | Yes |
| 124 | MPHOSPH9 |  |  | 644 | -0.384 | -0.6192 | Yes |
| 125 | TTF2 |  |  | 646 | -0.386 | -0.6187 | Yes |
| 126 | PHF19 |  |  | 648 | -0.391 | -0.6181 | Yes |
| 127 | TEDC1 |  |  | 651 | -0.394 | -0.6191 | Yes |
| 128 | POLE |  |  | 652 | -0.395 | -0.6169 | Yes |
| 129 | TMEM106C |  |  | 654 | -0.397 | -0.6163 | Yes |
| 130 | SHCBP1 |  |  | 655 | -0.398 | -0.6141 | Yes |
| 131 | GINS2 |  |  | 658 | -0.399 | -0.6151 | Yes |
| 132 | DCK |  |  | 659 | -0.400 | -0.6129 | Yes |
| 133 | CDT1 |  |  | 665 | -0.406 | -0.6187 | Yes |
| 134 | MNS1 |  |  | 666 | -0.408 | -0.6164 | Yes |
| 135 | SAE1 |  |  | 668 | -0.408 | -0.6158 | Yes |
| 136 | DSN1 |  |  | 671 | -0.410 | -0.6167 | Yes |
| 137 | NUDT1 |  |  | 674 | -0.413 | -0.6177 | Yes |
| 138 | NUP50 |  |  | 677 | -0.415 | -0.6186 | Yes |
| 139 | SAC3D1 |  |  | 679 | -0.416 | -0.6179 | Yes |
| 140 | PKMYT1 |  |  | 681 | -0.420 | -0.6171 | Yes |
| 141 | CCDC14 |  |  | 685 | -0.423 | -0.6196 | Yes |
| 142 | CENPO |  |  | 686 | -0.424 | -0.6173 | Yes |
| 143 | CDCA4 |  |  | 687 | -0.424 | -0.6149 | Yes |
| 144 | RPA2 |  |  | 688 | -0.424 | -0.6126 | Yes |
| 145 | CENPQ |  |  | 689 | -0.425 | -0.6102 | Yes |
| 146 | FANCA |  |  | 691 | -0.425 | -0.6094 | Yes |
| 147 | STIL |  |  | 692 | -0.425 | -0.6071 | Yes |
| 148 | NSD2 |  |  | 693 | -0.426 | -0.6047 | Yes |
| 149 | DIAPH3 |  |  | 696 | -0.427 | -0.6056 | Yes |
| 150 | CENPL |  |  | 703 | -0.432 | -0.6128 | Yes |
| 151 | CHEK1 |  |  | 704 | -0.433 | -0.6104 | Yes |
| 152 | PARPBP |  |  | 705 | -0.434 | -0.6080 | Yes |
| 153 | TIMELESS |  |  | 707 | -0.435 | -0.6072 | Yes |
| 154 | E2F1 |  |  | 708 | -0.435 | -0.6048 | Yes |
| 155 | POLD3 |  |  | 710 | -0.437 | -0.6040 | Yes |
| 156 | MZT1 |  |  | 711 | -0.437 | -0.6015 | Yes |
| 157 | ZNF93 |  |  | 712 | -0.438 | -0.5991 | Yes |
| 158 | LMNB2 |  |  | 713 | -0.438 | -0.5967 | Yes |
| 159 | RBBP8 |  |  | 714 | -0.440 | -0.5942 | Yes |
| 160 | RAD18 |  |  | 715 | -0.443 | -0.5918 | Yes |
| 161 | ALYREF |  |  | 716 | -0.444 | -0.5893 | Yes |
| 162 | SLC20A1 |  |  | 717 | -0.444 | -0.5868 | Yes |
| 163 | MASTL |  |  | 721 | -0.451 | -0.5892 | Yes |
| 164 | PCNT |  |  | 725 | -0.454 | -0.5915 | Yes |
| 165 | POLQ |  |  | 726 | -0.455 | -0.5889 | Yes |
| 166 | SPAG5 |  |  | 729 | -0.458 | -0.5896 | Yes |
| 167 | GINS1 |  |  | 730 | -0.459 | -0.5871 | Yes |
| 168 | POLA1 |  |  | 733 | -0.460 | -0.5877 | Yes |
| 169 | BRIP1 |  |  | 734 | -0.461 | -0.5852 | Yes |
| 170 | CSE1L |  |  | 736 | -0.461 | -0.5842 | Yes |
| 171 | C19orf48 |  |  | 738 | -0.462 | -0.5833 | Yes |
| 172 | E2F8 |  |  | 741 | -0.467 | -0.5839 | Yes |
| 173 | MGME1 |  |  | 742 | -0.468 | -0.5813 | Yes |
| 174 | SMC3 |  |  | 743 | -0.470 | -0.5787 | Yes |
| 175 | TK1 |  |  | 747 | -0.480 | -0.5808 | Yes |
| 176 | DDIAS |  |  | 748 | -0.482 | -0.5782 | Yes |
| 177 | MIS18A |  |  | 750 | -0.483 | -0.5771 | Yes |
| 178 | LIG1 |  |  | 752 | -0.485 | -0.5760 | Yes |
| 179 | PRR11 |  |  | 755 | -0.488 | -0.5765 | Yes |
| 180 | SPDL1 |  |  | 758 | -0.491 | -0.5770 | Yes |
| 181 | CDCA3 |  |  | 759 | -0.497 | -0.5742 | Yes |
| 182 | TRIP13 |  |  | 760 | -0.498 | -0.5715 | Yes |
| 183 | MMS22L |  |  | 761 | -0.500 | -0.5687 | Yes |
| 184 | MXD3 |  |  | 763 | -0.502 | -0.5675 | Yes |
| 185 | GINS4 |  |  | 765 | -0.510 | -0.5663 | Yes |
| 186 | GMNN |  |  | 766 | -0.510 | -0.5634 | Yes |
| 187 | RTKN2 |  |  | 767 | -0.511 | -0.5606 | Yes |
| 188 | LRR1 |  |  | 768 | -0.512 | -0.5578 | Yes |
| 189 | CMC2 |  |  | 771 | -0.518 | -0.5581 | Yes |
| 190 | CDC45 |  |  | 773 | -0.521 | -0.5568 | Yes |
| 191 | DTYMK |  |  | 774 | -0.522 | -0.5539 | Yes |
| 192 | PHTF2 |  |  | 775 | -0.523 | -0.5510 | Yes |
| 193 | RFC4 |  |  | 777 | -0.527 | -0.5497 | Yes |
| 194 | SKA3 |  |  | 783 | -0.539 | -0.5547 | Yes |
| 195 | NCAPD2 |  |  | 784 | -0.540 | -0.5517 | Yes |
| 196 | CEP152 |  |  | 785 | -0.542 | -0.5487 | Yes |
| 197 | BLM |  |  | 786 | -0.543 | -0.5457 | Yes |
| 198 | FANCD2 |  |  | 787 | -0.544 | -0.5427 | Yes |
| 199 | RPA3 |  |  | 788 | -0.550 | -0.5396 | Yes |
| 200 | NCAPD3 |  |  | 790 | -0.559 | -0.5381 | Yes |
| 201 | ATAD5 |  |  | 791 | -0.559 | -0.5350 | Yes |
| 202 | HAUS8 |  |  | 792 | -0.560 | -0.5319 | Yes |
| 203 | RACGAP1 |  |  | 796 | -0.566 | -0.5336 | Yes |
| 204 | ASF1B |  |  | 798 | -0.568 | -0.5321 | Yes |
| 205 | CENPJ |  |  | 799 | -0.568 | -0.5289 | Yes |
| 206 | HES6 |  |  | 800 | -0.569 | -0.5258 | Yes |
| 207 | RFC3 |  |  | 801 | -0.569 | -0.5226 | Yes |
| 208 | CEP78 |  |  | 803 | -0.570 | -0.5210 | Yes |
| 209 | CENPN |  |  | 805 | -0.574 | -0.5194 | Yes |
| 210 | USP1 |  |  | 806 | -0.575 | -0.5163 | Yes |
| 211 | CDC6 |  |  | 807 | -0.584 | -0.5130 | Yes |
| 212 | CDCA8 |  |  | 808 | -0.585 | -0.5098 | Yes |
| 213 | CHAF1A |  |  | 810 | -0.588 | -0.5081 | Yes |
| 214 | PSMC3IP |  |  | 811 | -0.588 | -0.5048 | Yes |
| 215 | FOXM1 |  |  | 814 | -0.593 | -0.5048 | Yes |
| 216 | DEPDC1 |  |  | 815 | -0.596 | -0.5014 | Yes |
| 217 | MCM7 |  |  | 817 | -0.599 | -0.4997 | Yes |
| 218 | LMNB1 |  |  | 818 | -0.606 | -0.4964 | Yes |
| 219 | MCM8 |  |  | 819 | -0.607 | -0.4930 | Yes |
| 220 | MND1 |  |  | 821 | -0.615 | -0.4912 | Yes |
| 221 | CKAP2L |  |  | 826 | -0.625 | -0.4942 | Yes |
| 222 | CCDC34 |  |  | 827 | -0.626 | -0.4907 | Yes |
| 223 | DNAJC9 |  |  | 828 | -0.633 | -0.4872 | Yes |
| 224 | AURKB |  |  | 829 | -0.634 | -0.4836 | Yes |
| 225 | HELLS |  |  | 830 | -0.635 | -0.4801 | Yes |
| 226 | DTL |  |  | 831 | -0.637 | -0.4766 | Yes |
| 227 | PCNA |  |  | 832 | -0.640 | -0.4730 | Yes |
| 228 | PIMREG |  |  | 833 | -0.641 | -0.4694 | Yes |
| 229 | BUB3 |  |  | 834 | -0.641 | -0.4659 | Yes |
| 230 | CENPW |  |  | 835 | -0.643 | -0.4623 | Yes |
| 231 | CDKN3 |  |  | 836 | -0.653 | -0.4587 | Yes |
| 232 | BUB1 |  |  | 837 | -0.653 | -0.4551 | Yes |
| 233 | ITGB3BP |  |  | 838 | -0.661 | -0.4514 | Yes |
| 234 | E2F7 |  |  | 840 | -0.675 | -0.4492 | Yes |
| 235 | KIF22 |  |  | 842 | -0.678 | -0.4471 | Yes |
| 236 | BIRC5 |  |  | 843 | -0.679 | -0.4433 | Yes |
| 237 | CENPM |  |  | 844 | -0.682 | -0.4395 | Yes |
| 238 | NCAPH |  |  | 845 | -0.685 | -0.4357 | Yes |
| 239 | BRCA1 |  |  | 846 | -0.691 | -0.4319 | Yes |
| 240 | ZWINT |  |  | 847 | -0.692 | -0.4280 | Yes |
| 241 | SPC24 |  |  | 848 | -0.694 | -0.4242 | Yes |
| 242 | CENPH |  |  | 849 | -0.694 | -0.4203 | Yes |
| 243 | BRCA2 |  |  | 850 | -0.694 | -0.4165 | Yes |
| 244 | DHFR |  |  | 851 | -0.701 | -0.4126 | Yes |
| 245 | VRK1 |  |  | 852 | -0.711 | -0.4086 | Yes |
| 246 | CIP2A |  |  | 853 | -0.712 | -0.4047 | Yes |
| 247 | ECT2 |  |  | 854 | -0.717 | -0.4007 | Yes |
| 248 | KIF2C |  |  | 855 | -0.721 | -0.3967 | Yes |
| 249 | NUF2 |  |  | 856 | -0.721 | -0.3927 | Yes |
| 250 | CENPK |  |  | 857 | -0.722 | -0.3887 | Yes |
| 251 | FAM111A |  |  | 858 | -0.727 | -0.3846 | Yes |
| 252 | SYNE2 |  |  | 859 | -0.728 | -0.3806 | Yes |
| 253 | ANLN |  |  | 860 | -0.733 | -0.3765 | Yes |
| 254 | KIF4A |  |  | 861 | -0.736 | -0.3724 | Yes |
| 255 | FEN1 |  |  | 862 | -0.736 | -0.3683 | Yes |
| 256 | HMMR |  |  | 863 | -0.740 | -0.3642 | Yes |
| 257 | FBXO5 |  |  | 864 | -0.742 | -0.3601 | Yes |
| 258 | CCNB2 |  |  | 865 | -0.745 | -0.3559 | Yes |
| 259 | RRM1 |  |  | 866 | -0.749 | -0.3518 | Yes |
| 260 | ARHGAP11A |  |  | 867 | -0.756 | -0.3476 | Yes |
| 261 | NDC80 |  |  | 869 | -0.762 | -0.3450 | Yes |
| 262 | ORC6 |  |  | 870 | -0.766 | -0.3407 | Yes |
| 263 | BUB1B |  |  | 871 | -0.766 | -0.3364 | Yes |
| 264 | CCNA2 |  |  | 872 | -0.770 | -0.3322 | Yes |
| 265 | KIF23 |  |  | 873 | -0.772 | -0.3279 | Yes |
| 266 | MELK |  |  | 874 | -0.776 | -0.3236 | Yes |
| 267 | TMPO |  |  | 875 | -0.778 | -0.3192 | Yes |
| 268 | KIF20B |  |  | 876 | -0.779 | -0.3149 | Yes |
| 269 | CEP55 |  |  | 877 | -0.780 | -0.3106 | Yes |
| 270 | CKAP2 |  |  | 878 | -0.781 | -0.3062 | Yes |
| 271 | NCAPG2 |  |  | 879 | -0.781 | -0.3019 | Yes |
| 272 | PTTG1 |  |  | 880 | -0.791 | -0.2975 | Yes |
| 273 | MCM10 |  |  | 881 | -0.798 | -0.2931 | Yes |
| 274 | H2AFX |  |  | 882 | -0.799 | -0.2886 | Yes |
| 275 | KIFC1 |  |  | 883 | -0.807 | -0.2841 | Yes |
| 276 | KIF14 |  |  | 885 | -0.809 | -0.2813 | Yes |
| 277 | EZH2 |  |  | 886 | -0.810 | -0.2768 | Yes |
| 278 | UBE2S |  |  | 887 | -0.815 | -0.2722 | Yes |
| 279 | FAM111B |  |  | 888 | -0.822 | -0.2677 | Yes |
| 280 | SGO1 |  |  | 889 | -0.822 | -0.2631 | Yes |
| 281 | KIF15 |  |  | 890 | -0.834 | -0.2585 | Yes |
| 282 | CDCA5 |  |  | 892 | -0.841 | -0.2554 | Yes |
| 283 | RAD51AP1 |  |  | 894 | -0.853 | -0.2523 | Yes |
| 284 | KPNA2 |  |  | 895 | -0.858 | -0.2475 | Yes |
| 285 | DBF4 |  |  | 896 | -0.864 | -0.2427 | Yes |
| 286 | SMC2 |  |  | 897 | -0.867 | -0.2379 | Yes |
| 287 | SGO2 |  |  | 898 | -0.869 | -0.2330 | Yes |
| 288 | PBK |  |  | 900 | -0.880 | -0.2298 | Yes |
| 289 | FANCI |  |  | 901 | -0.882 | -0.2249 | Yes |
| 290 | SMC4 |  |  | 902 | -0.886 | -0.2199 | Yes |
| 291 | MIS18BP1 |  |  | 904 | -0.889 | -0.2166 | Yes |
| 292 | C21orf58 |  |  | 905 | -0.893 | -0.2117 | Yes |
| 293 | CCNE2 |  |  | 907 | -0.895 | -0.2083 | Yes |
| 294 | XRCC2 |  |  | 908 | -0.907 | -0.2032 | Yes |
| 295 | CKS1B |  |  | 909 | -0.912 | -0.1982 | Yes |
| 296 | HJURP |  |  | 910 | -0.921 | -0.1931 | Yes |
| 297 | CKS2 |  |  | 911 | -0.922 | -0.1879 | Yes |
| 298 | GTSE1 |  |  | 912 | -0.941 | -0.1827 | Yes |
| 299 | KIF11 |  |  | 913 | -0.957 | -0.1774 | Yes |
| 300 | MYBL2 |  |  | 914 | -0.959 | -0.1721 | Yes |
| 301 | KNL1 |  |  | 915 | -0.963 | -0.1667 | Yes |
| 302 | CLSPN |  |  | 916 | -0.964 | -0.1614 | Yes |
| 303 | TYMS |  |  | 917 | -0.965 | -0.1560 | Yes |
| 304 | CENPU |  |  | 918 | -0.971 | -0.1506 | Yes |
| 305 | PRC1 |  |  | 919 | -0.980 | -0.1452 | Yes |
| 306 | MAD2L1 |  |  | 921 | -0.990 | -0.1413 | Yes |
| 307 | UBE2T |  |  | 922 | -0.994 | -0.1357 | Yes |
| 308 | SPC25 |  |  | 924 | -1.033 | -0.1316 | Yes |
| 309 | HIST1H1E |  |  | 925 | -1.049 | -0.1258 | Yes |
| 310 | ESCO2 |  |  | 926 | -1.067 | -0.1198 | Yes |
| 311 | UBE2C |  |  | 928 | -1.108 | -0.1153 | Yes |
| 312 | NUSAP1 |  |  | 929 | -1.112 | -0.1091 | Yes |
| 313 | TPX2 |  |  | 930 | -1.124 | -0.1029 | Yes |
| 314 | NCAPG |  |  | 931 | -1.142 | -0.0965 | Yes |
| 315 | ASPM |  |  | 932 | -1.146 | -0.0902 | Yes |
| 316 | ATAD2 |  |  | 933 | -1.162 | -0.0837 | Yes |
| 317 | CENPF |  |  | 935 | -1.200 | -0.0787 | Yes |
| 318 | HMGB2 |  |  | 937 | -1.238 | -0.0734 | Yes |
| 319 | CDK1 |  |  | 938 | -1.248 | -0.0665 | Yes |
| 320 | RRM2 |  |  | 940 | -1.403 | -0.0603 | Yes |
| 321 | MKI67 |  |  | 941 | -1.407 | -0.0524 | Yes |
| 322 | TOP2A |  |  | 942 | -1.417 | -0.0446 | Yes |
| 323 | PCLAF |  |  | 943 | -1.554 | -0.0359 | Yes |
| 324 | HIST1H1D |  |  | 944 | -1.810 | -0.0259 | Yes |
| 325 | HIST1H1B |  |  | 945 | -1.903 | -0.0153 | Yes |
| 326 | HIST1H4C |  |  | 946 | -2.752 | -0.0000 | Yes |
Table: GSEA details [plain text format]

  

Fig 2: DOROSZKO\_U3054MG\_CLUSTER\_4: Random ES distribution      
 Gene set null distribution of ES for **DOROSZKO\_U3054MG\_CLUSTER\_4**
